# Systematic review of Mendelian randomization studies on Parkinson’s disease

**DOI:** 10.1515/medgen-2022-2139

**Published:** 2022-08-12

**Authors:** Sophia Kappen, Daniele Bottigliengo, Amke Caliebe, Fabiola Del Greco M., Inke R. König

**Affiliations:** Institute of Medical Biometry and Statistics, University of Lübeck, University Hospital Schleswig-Holstein, Ratzeburger Allee 160, 23562 Lübeck, Germany; Institute for Biomedicine, Eurac Research, Via Galvani 31, 39100 Bolzano, Italy; Institute of Medical Informatics and Statistics, University Hospital Schleswig-Holstein, Kiel, Germany; Kiel University, Kiel, Germany

**Keywords:** Mendelian randomization, causal inference, Parkinson’s disease, systematic review, risk factors

## Abstract

**Background:**

Parkinson‘s disease (PD) is known to be associated with non-genetic factors. To infer causality, Mendelian randomization (MR) studies are increasingly used. Here, genetic variants are used as instrumental variables for the risk factor but have no direct effect on PD themselves.

**Methods:**

We performed a systematic literature review on MR studies for PD. Studies were identified searching the PubMed database. Upon data extraction, we evaluated the methodological quality and summarized the evidence.

**Results:**

Twelve articles were included. Most studies showed “good” methodological quality, but most did not report proper power estimations. Twelve analyses yielded nominally significant effects.

**Conclusions:**

Our systematic review shows that most MR studies were well performed and allow to identify causal exposures, which may inform further studies on the prevention and early intervention of PD.

## Introduction

Parkinson‘s disease (PD) is a complex neurodegenerative disorder, where the diagnosis is mostly based on clinical motor and non-motor symptoms [1], [2], [3]. Following diagnosis, currently available treatment options for PD are symptomatic only, because the understanding of the pathobiology and etiology of PD is still limited. Specifically, a number of genetic factors are known including variants in monogenic disease genes as well as about 90 genetic risk variants associated with PD. In addition, modifiable environmental risk factors and lifestyle exposures have been suggested based on observed associations [4], [5], [6], [7], [8], [9]; but as yet, the causality of these is mostly unknown since, instead of resulting from causation, these associations could also be the result of reverse causation, confounding, or selection bias, among others. Proving causality could open additional paths for the development of preventive or therapeutic strategies. Randomized clinical trials (RCTs), however, which are the gold stardard for investigating causality, are not feasible for many of these factors.

Among different study designs, Mendelian randomization (MR) represents a statistical approach to infer causality of a modifiable exposure for a disease, using genetic variants strongly associated with the exposure as instrumental variable [10]. MR is an approach that mimics an RCT, using the random allocation of the alleles of the genetic instrument and reproducing the blinding conditions, because probands’ genetics are usually not known by themselves or by medical doctors. Thus, it overcomes typical limitations of observational studies, that is confounding and reverse causation [9]. At the same time, it has distinct advantages over RCTs that may not be ethically or biologically feasible for the type of exposure or outcome or simply too expensive. Another key feature is that MR estimates the effect of life-long exposures as opposed to usually limited time frames in RCTs. However, the reliability and the robustness of a MR study rely on three assumptions that need be fulfilled by the genetic instrument (Supplementary Figure S1): i. To be strongly associated with the risk factor of interest (relevance); ii. not to be associated with unmeasured confounders of the risk factor and the disease, i. e. it does not share any causes with the outcome (exchangeability); iii. not to be directly associated with the disease outcome (exclusion restriction, absence of pleiotropy) [11]. These assumptions need to be checked in any MR study for valid conclusions.

Starting with the investigation of the causality of serum iron levels in 2013 [12], MR has become popular over the past decade also in the context of PD. This systematic review therefore aimed to identify and summarise causal findings related to PD obtained from MR studies. After summarising the current evidence of causality on PD, we evaluate the robustness of those results considering the strengths and limitations of the studies.

**Figure 1:**
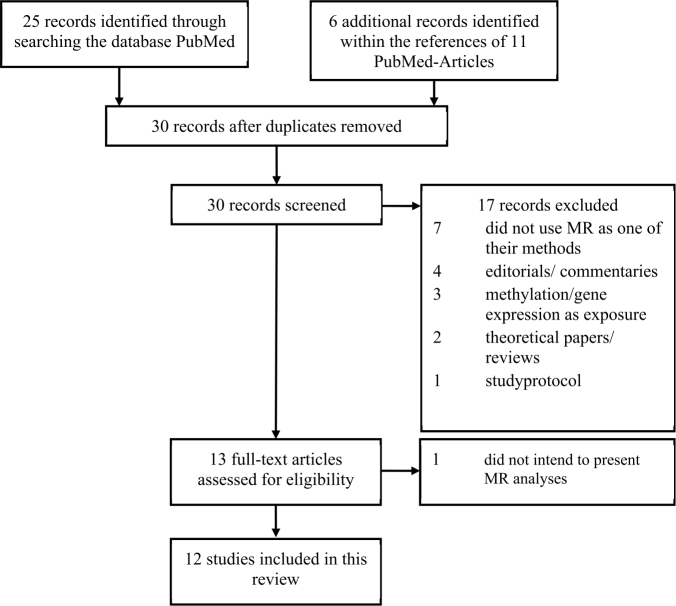
Workflow of the literature search.

## Methods

### Search strategy and inclusion criteria

MR studies exploring the causality of any risk or protective factor on PD were identified searching the PubMed database (https://pubmed.ncbi.nlm.nih.gov/) in January 2021, with no date, language or ethnicity restrictions, following the PRISMA (Preferred Reporting Items for Systematic Reviews and Meta-analysis) guidelines [13] and using the following search term: ((analysis, mendelian randomization[MeSH Terms]) OR (mendelian randomization analysis[MeSH Terms]) OR (mendelian randomization[TIAB]) OR (mendelian randomisation[TIAB]) OR (genetic instrument[TIAB]) OR (instrumental variable[TIAB])) AND ((parkinson’s disease[MeSH Terms]) OR (parkinson disease[MeSH Terms])). Abstracts of all articles were screened for relevance. Only original research articles applying MR were retained, and articles were excluded if methylation and/or gene expression was investigated as exposures, if PD was not the outcome, or if the specific purpose of the article was not to present MR analyses. In addition, references of the identified articles were searched for further relevant articles. The workflow of the literature search is described in Figure 1. One author (SK) screened titles and abstracts for eligibility and a second author (IRK) independently screened a random sample. Discrepancies were resolved by consensus between the two authors.

**Table 1:** Extraction form.

**Article details**	Authors
	Title
	Reference
	Exposure(s)
	Special features
	Data source: one sample/two samples
	Two samples are independent: Yes/No/NA
	Sample size(s) – Exposure
	Sample size(s) – PD: n(cases), n(controls)
	Ethnicity
**Genetic instruments**	Number of SNPs in instrument: N/unspecified
	Criteria for SNPs selection: p-value association/assumed function/other
	Independent SNPs: yes/no/unspecified; specify r^2^ if given
**Core MR assumptions**	Check of MR-I: F statistic/R^2^/other; unspecified
	Check of MR-II: confounders tested (list)/unspecified
	Check of MR-II discussed: yes/no
	Check of MR-III (horizontal pleiotropy investigated): yes/no
	The authors exclude possible pleiotropic instruments due to previous literature results: yes/no
**Method reporting**	Main method for drawing conclusions: IVW/MR Egger/weighted median or mode/two-stage least square/Wald ratio estimate/other
	Additional analyses performed
**Data presentation**	Authors present the results: yes/no
	Authors calculate an observational estimate on their own sample: yes/no
	Authors provide the data that they used in a supplement to allow researchers to reproduce their findings: yes/no/partly
**Results**	Provided results: OR/HR and CI; unspecified
	Evidence of causality: yes/no/unspecified
**Power estimation**	Power calculation: yes/no; if yes: estimated value (only OR >1 reported, otherwise 1/OR)
**Interpretation**	Clinical interpretation of the causal estimates provided: yes/no/unspecified
**Clinical implications**	Clinical trials evidence provided/discussed:yes/no

**Table 2:** Score system for evaluation of methodological quality.

Domain	Question	Answer	Score
***1. Study details***	In the two-sample setting, are the two samples independent or do the authors mention the possible overlap between gene-exposure and gene-PD associations? In the one-sample setting, are appropriate estimation methods used?	Yes/No	1/0
***2. Genetic instruments’ characteristics***	Are the analyses restricted to independent instruments or do the analyses allow for the correlation between them? Do the authors mention the issue of instruments’ correlation?	Yes/No	1/0
***3. MR assumption I***	Do the authors check at least partially the 1st assumption on the instruments?	Yes/No	1/0
***4. MR assumption II***	Do the authors check or discuss at least partially the 2nd assumption on the instruments?	Yes/No	1/0
***5. MR assumption III***	Do the authors check or discuss at least partially the 3rd assumption on the instruments?	Yes/No	1/0
***6. Reporting methods***	Are conclusions drawn from one main method?	Yes/No	1/0
***7. Results evaluation***	Do the authors provide sensitivity analyses or observational estimates?	Yes/No	1/0
***8. Reporting results***	Do the authors provide estimates and confidence intervals?	Yes/No	1/0
***9. Power estimation***	Are power calculations performed or mentioned?	Yes/No	1/0
***10. Results interpretation***	Is clinical interpretation of the causal estimates provided?	Yes/No	1/0

### Data extraction

From the identified articles, data were extracted in duplicate (SK and AC) using a data extraction form (Table 1) based on a published checklist for evaluating MR studies [14]. If the analysis of more than one exposure was reported, details were extracted for every analysis separately.

Disagreements or ambiguities were resolved through consensus and, when necessary, through discussion with all co-authors.

### Quality score assessment

The methodological quality of the identified MR studies was assessed using a score derived from methodological considerations [15]. For this, ten key questions were considered from the extraction form, and a score was attributed to each of them (Table 2). Every question targets a possible methodological issue that should be addressed or at least discussed. Two answers are possible: (1), if the issue was addressed or at least discussed; (0), if the issue was neither addressed nor discussed. The total score ranges from 0 (“Poor study”) to 10 (“Excellent study”). Intermediate scores are “Very good”, for scores 9–8; “Good”, for score 7; “Fair”, for score 6; “Weak”, for scores 5–4; and “Mediocre”, for scores 3–1. When multiple MR analyses were reported within one article, we assigned one overall score to the article, since the same workflow was applied to each analysis.

## Statistical analyses

The strength of the genetic instruments and the power of the MR analyses were evaluated using the variance explained by the instruments (R^2^). When R^2^ was not given in the article, it was calculated using the formula R^2^ = F/(N −2 + F), where F is the reported F-statistic and N is the sample size of the gene-exposure association [12], [16]. Starting from the extracted study characteristics (R^2^ values and sample sizes), we provide an overview of the power, using a non-centrality parameter-based approach [17] implemented in the publically available tool mRnd (https://shiny.cnsgenomics.com/mRnd/), for a type I error rate of 0.05.

Causal effects were extracted for every analysis together with confidence intervals. In the article of Nalls et al. [7] a 95 % confidence interval was not available and it was calculated from the corresponding effect and standard error estimates. Causal associations are described graphically. A two-sided p-value of 0.05 was considered nominally significant.

All statistical analyses were conducted with the software Rstudio version 4.1.0 (https://www.R-project.org/).

**Table 3:** Overall quality evaluation for each article.

Study	Total score
Benn et al. (2017)	8
Cheng et al. (2019)	6
Fang et al. (2019)	7
Grover et al. (2019)	9
Kia et al. (2018)	10
Kobylecki et al. (2018)	10
Larsson et al. (2017)	9
Nalls et al. (2019)	5
Noyce et al. (2017)	10
Pichler et al. (2013)	9
Prins et al. (2016)	9
Simon et al. (2014)	8

## Results

The results of the literature search process are shown in Figure 1. After screening title and abstract of 30 identified records, 12 articles were included in the systematic review, of which four reported more than one MR analysis, because multiple exposures had been investigated. Hence, the total number of MR analyses was 27.

### Quality evaluation

The scoring system was applied to the 12 selected articles, and overall results are presented in Table 3 with details for every question given in Supplementary Table S2. According to the scoring system, no studies with “poor” quality were reported, with scores always greater than 4. Overall, 75 % of the studies were classified as at least “good”. In detail, three (25 %) of these studies were rated as “excellent”, five (42 %) as “very good”, one (8 %) as “good”, one (8 %) as “fair”, and one (8 %) as “weak”. Note that in the article of [8], which was classified as weak, MR was not the focus of the study. Instead the study was primarily a large meta GWAS for PD. As shown in the Supplementary Figure S2, going through the ten key methodological questions, all MR articles were well designed in terms of awareness of the possible sources of bias due to overlapping gene-exposure and gene-PD association studies. Results were also well-reported and evaluated. However, the majority of the articles did not report proper power estimations.

### Descriptive statistics

Data extracted for each MR study are reported in Supplementary Table S1. Among the selected analyses, there were three one-sample MR analyses [18], [19], [20] and 24 two-sample MR analyses. Five MR analyses were on binary exposures [7], [21]; two on categorical exposures with more than 2 categories [7], [21] (Supplementary Table S3), and the remaining 20 were on continuous ones. Except for the article reporting the analysis of multiple exposures where both gene-exposure and gene-PD estimates came from a mixed population [22], all of them were carried out in data from European ancestry populations.

One-sample studies were smaller than two-sample studies. Indeed, the former were characterized by the inclusion of a small number of PD cases (460; 279; 735). In contrast, the latter reported case numbers ranging from 5,333 to 38,426. The sample sizes of the gene-exposure associations varied from 2,600 to 939,908 for all analyses.

All MR analyses included multiple SNPs as instruments, with numbers ranging from 2 to 244. Ten of them used up to 10 SNPs, five used 11 to 77 SNPs, and seven were based on hundreds of SNPs (from 109 to 244). Only the meta analysis of Nalls and colleagues [7] with multiple exposures did not specify the number of instruments. Moreover, the authors of this article did not provide information about the strength of the instrument and did not discuss the possible violation of the three core MR assumptions. Regarding a check of the linkage disequilibrium of the SNPs, two articles provided no information, and in further two articles, it was reported that instruments were independent without specifying values. Six articles described the r^2^ threshold or range, and in the remaining two articles, the selection of instruments was performed after clumping SNPs using both the r^2^ and the bp distance between variants: 10000 kb [9]; 140 kb [23].

Only one article reporting five analyses used a polygenic risk score [7]. Prins et al. constructed two instruments, one comprised of significantly associated SNPs, the other of SNPs only within the functional gene [24].

Regarding the strength of the instruments, six of the articles reported the F-statistic value, three verified the first MR assumption otherwise and three gave no information about the strength of the instruments. Where the F value is given, all studies used strong instruments (mean F > 10) [25].

The second MR assumption was checked or discussed in nine articles. The presence of pleiotropy was investigated in eleven articles. The majority of them performed the heterogeneity Cochran Q test with the I^2^ index [26] or performed additional robust MR analyses. In three articles, pleiotropic effects were explored by checking the direct involvement of SNPs in PD with a literature search for pathogenesis, by using a stepwise removal of SNPs with a potential pleiotropic effect based on a single Cochran Q value or by selecting SNPs located only in the functional gene.

### Causal effects estimation and clinical interpretation

In the two-sample MR studies, the main method applied for exploring causality was the inverse-variance weighted (IVW) method. One paper, however, reported no main method, but applied equally IVW, MR Egger and weighted median [22]. In the one-sample MR studies, the used methods were the generalised method of moments [18], generalized linear models [20], and control function instrumental variable estimator [19]. Except for Cheng and colleagues [22], all additional methods were applied to explore the presence of pleiotropy.

Causal estimates (odds ratio, log(odds ratio), risk ratio or hazard ratio) were provided for all the MR analyses (Supplementary Table S3), and all of them were compared with conventional observational estimates; the latter had been obtained using individual data from the same study only in four MR studies [18], [19], [20], [27].

The 95 % confidence intervals were provided for all the analyses, except for one that reported OR and standard error [7].

**Figure 2:**
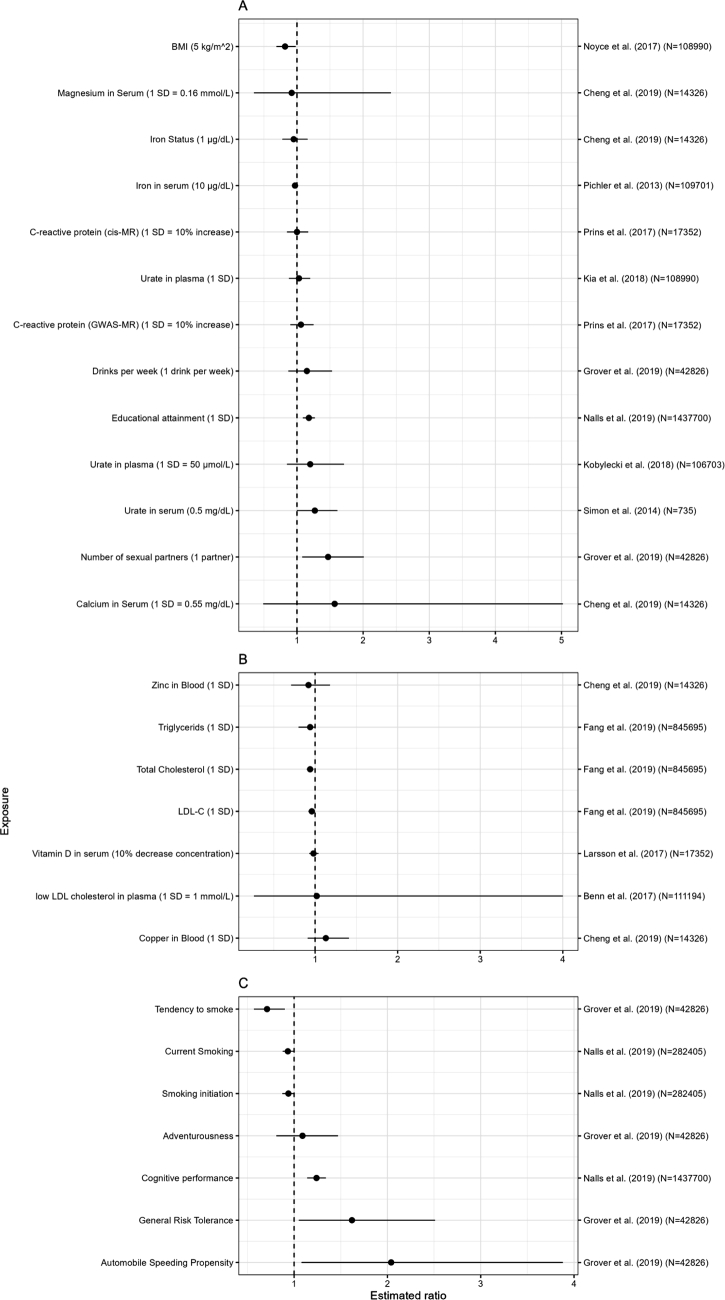
Forest plots by exposure. On the left side, the names of the exposures are shown along with the increase used to express their effect on the outcomes (1 SD is a one standard deviation increase in the exposure). The reference article along with the sample size of the outcome data for each exposure are shown on the right side of the plot. On the x-axis, the effect measure, i. e. Odds Ratio (OR), Relative Risk (RR), or Hazard Ratio (HR), termed as “Estimate Ratio”, are depicted. The dots represent the point estimate of the effect measure and the line the width of the 95 % CIs.

Out of the 27 investigated exposures, 12 were nominally significant, with a two-sided p-value smaller than 0.05 (Supplementary Figure S3). As shown in Figure 2, half of those had protective effects (total cholesterol; LDL-cholesterol; tendency to smoke; smoking initiation; body mass index; iron), and the other half had risk effects (general risk tolerance; automobile speed propensity; number of sexual partners; cognitive performance; educational attainment; urate in serum). The reported risk reduction varied from 4 % (LDL-C) to 29 % (tendency to smoke), and the reported risk increase varied from 18 % (educational attainment) to 104 % (automobile speed propensity).

A clinical interpretation was presented in nine articles. Lastly, for the nine two-sample articles, genetic summary data used in the MR analysis were made directly available in five articles, partly available in three articles, and only one article provided no data at all. The individual genotype data of the three one-sample articles are not openly accessable.

### Power analysis

Power estimations were provided in six articles [9], [18], [19], [21], [23], [28]. Among these, the following exposures were found not to be causally associated with PD: LDL cholesterol in plasma [18]; adventurousness; drinks per week [18]; urate in plasma [23]; vitamin D in serum [28]. Noyce et al. reported a power of 85 % for the observed significant OR of 0.82 for BMI (significance level 5 %). In the study of Kobylecki et al. [28] only a power of 19 % was obtained for an OR of 1.39 when investigating the influence of urate in plasma, a non-significant result.

An overview of the power is provided using sample sizes from 20,000 to 100,000, variance explained levels R^2^ from 0.01 % to 5 %, ORs values from 1.02 to 1.3, and cases proportions from 0.1 to 0.2 from the performed MR studies on PD (Supplementary Figure S4). Although some studies were carried out on large datasets, our results indicate that the power was very low for scenarios with small effects and weak instruments.

## Discussion

The present systematic review on MR studies on PD showed that 10 exposures out of 27 could be considered as potential causal exposures after correction for multiple testing. When interpreting the effect sizes, it should be kept in mind that in MR studies, the lifetime effect of the exposure is estimated, given that the effect of the genetic variants on the exposure is assumed to be constant across the life course. Thus, MR leads to typically larger estimates compared with those obtained with alternative studies. In particular, it was found that body mass index and iron could have a dominant role in the prevention of the disease, given that they were shown to decrease the risk of PD by 18 % per 5 BMI points and 12 % per 10 mg/dl iron, respectively [8], [9]. Additionally, a lower risk was conferred by higher levels of TC and LDL-C [8], [9]. Moreover, an MR analysis on “tendency to smoke” (smokers versus no smokers) also suggested a PD risk reduction of 29 % for smokers [21], confirming an inverse association identified by many observational studies. Among potential causal risk factors, serum urate could represent an important biomarker to take under control [20]. Educational attainment, cognitive performance and risk behaviours like general risk tolerance could increase the PD risk as well [7], [21], although the possibly underlying pathways are probably more intricate and require more in-depth studies.

It should be noted that, when investigating several potential risk factors, not all articles reported to have corrected for multiple testing. Thus, associations reported to be significant should be viewed with caution. The correction for multiple testing in this context, however, is intricate. First, in addition to investigating more than one risk factor, often multiple analysis strategies, multiple outcomes and/or multiple instruments are used, which will yield correlated results but should be accounted for. Second, the by now vast number of genetic datasets for potential risk factors invites the analysis of a multitude of associations without strong a priori plausibility, thus likely increasing the percentage of false positive results if not interpreted carefully. Transparent reports of all investigated associations, including those with non-significant results, are therefore especially important.

Regarding the investigated fifteen exposures that were found not to be causally associated with PD, nine did not report power calculations. Reverse causation was not investigated in any study, meaning that PD as the cause for varying levels of an exposure was not considered. Importantly, given the disease complexity, evidence of causality of a biomarker on PD does not exclude any effect of the disease, or its progression, on the same biomarker, which is thus an interesting topic to pursue in future studies. The presence of pleiotropy is another important issue in MR analyses that is not straightforward to control. The presence of pleiotropy was discussed in all of the studies. Indeed, pleiotropic effects lead to biased estimates towards the null [26], and robust methods might underestimate the causal effect, increasing the number of false negatives.

Overall, our quality evaluation found that most of the studies were well performed following the established MR standards. We would like to point out that more in-depth assessments of the studies are possible using most recent guidelines on the performance and reporting of MR studies [29], [30]. However, these are not designed to give an overall evaluation of research quality [31], which is why we used a score derived from methodological considerations to mirror the general impression of the methodological quality.

Finally, it can be discussed to what extent our systematic review was affected by publication bias. In our experience, currently usually both positive (with causal evidence) and negative (without causal evidence) MR studies are published, thus making a publication bias unlikely. This is also reflected in the reviewed studies here, where only 12 of the 27 analyses showed a significant result. Moreover, those results could be reproduced and replicated, because the majority of the studies were performed using summary public data or provided the used datasets.

In conclusion, our first systematic review on MR studies shows the potential of this design to identify causal exposures, which may in turn inform further studies on the prevention and early intervention of PD.

## Supplementary Material

Supplementary figures

Supplementary tables
