# Supplementary material for: Systematic review of Mendelian randomization studies on Parkinson’s disease
Source: Med Genet. 2022 Aug 12;34(2):143–50. doi: 10.1515/medgen-2022-2139 (PMC11006297; doi:10.1515/medgen-2022-2139)

Supplementary material

Supplementary tables

Table S1: Data extracted for all the analyses for every article (excel file)

Table S2: Quality evaluation scores for each article (excel file)

Table S3: Summary information for each exposure.

Study	Exposure	Type	N (exposure data)	N (PD data)	Effect measure	Estimate (95% CI)
Benn et al. (2017)	low LDL cholesterol in plasma (1 SD = 1 mmol/L)	continuous	111,194	111,194	RR	1.02 (0.26; 4.00)
Cheng et al. (2019)	Calcium in serum (1 SD = 0.55 mg/dL)	continuous	39,400	14,326	OR	1.57 (0.49; 5.02)
Cheng et al. (2019)	Magnesium in serum (1 SD = 0.16 mmol/L)	continuous	15,366	14,326	OR	0.92 (0.35; 2.42)
Cheng et al. (2019)	Iron status (1 µg/dL)	continuous	48,972	14,326	OR	0.95 (0.78; 1.16)
Cheng et al. (2019)	Copper in blood (1 SD)	continuous	2,603	14,326	OR	1.13 (0.91; 1.41)
Cheng et al. (2019)	Zinc in blood (1 SD)	continuous	2,603	14,326	OR	0.92 (0.71; 1.18)
Fang et al. (2019)	Total cholesterol (1 SD)	continuous	188,577	845,695	OR	0.94 (0.9; 0.98)
Fang et al. (2019)	LDL-C (1 SD)	continuous	188,577	845,695	OR	0.96 (0.92; 0.99)
Fang et al. (2019)	Triglycerids (1 SD)	continuous	188,577	845,695	OR	0.94 (0.8; 1)
Grover et al. (2019)	General risk tolerance	categorical (binary)	939,908	42,826	OR	1.62 (1.05; 2.51)
Grover et al. (2019)	Adventurousness	categorical (binary)	557,923	42,826	OR	1.09 (0.81; 1.47)
Grover et al. (2019)	Automobile speeding propensity	categorical	404,291	42,826	OR	2.04 (1.08; 3.88)
Grover et al. (2019)	Drinks per week (1 drink per week)	continuous	414,343	42,826	OR	1.15 (0.87; 1.53)
Grover et al. (2019)	Tendency to smoke	categorical (binary)	518,633	42,826	OR	0.71 (0.57; 0.90)
Grover et al. (2019)	Number of sexual partners (1 partner)	continuous	370,711	42,826	OR	1.47 (1.08; 2.01)
Kia et al. (2018)	Urate in plasma (1 SD)	continuous		108,990	OR	1.03 (0.88; 1.20)
Kobylecki et al. (2018)	Urate in plasma (1 SD = 50 µmol/L)	continuous	106,703	106,703	OR	1.2 (0.85; 1.71)
Larsson et al. (2017)	Vitamin D in serum (10% decrease concentration)	continuous	42,024	17,352	OR	0.98 (0.93; 1.04)
Nalls et al. (2019)	Cognitive performance	categorical	257,828	1,437,700	OR	1.24 (1.14; 1.34)
Nalls et al. (2019)	Educational attainment (1 SD)	continuous	766,345	1,437,700	OR	1.18 (1.09; 1.27)

Study	Exposure	Type	N (exposure data)	N (PD data)	Effect measure	Estimate (95% CI)
Nalls et al. (2019)	Current smoking	categorical (binary)	47,419	282,405	OR	0.93 (0.88; 0.99)
Nalls et al. (2019)	Smoking initiation	categorical (binary)	208,988	282,405	OR	0.94 (0.88; 1.00)
Noyce et al. (2017)	BMI (1SD = 5 kg/m^2)	continuous	339,224	108,990	OR	0.82 (0.69; 0.98)
Pichler et al. (2013)	Iron in serum (10 µg/dL)	continuous	21,567	109,701	OR	0.97 (0.94; 0.99)
Prins et al. (2017)	C-reactive protein (cis-MR) (1 SD = 10% increase)	continuous	123,865	17,352	OR	1.00 (0.85; 1.17)
Prins et al. (2017)	C-reactive protein (GWAS-MR) (1 SD = 10% increase)	continuous	123,865	17,352	OR	1.06 (0.90; 1.25)
Simon et al. (2014)	Urate in serum (0.5 mg/dL)	continuous	735	735	HR	1.27 (1.00; 1.61)

Supplementary figures

Figure S1: Mendelian randomization causal graph. IV1: First MR assumption; IV2: Second MR assumption; IV3: Third MR assumption.

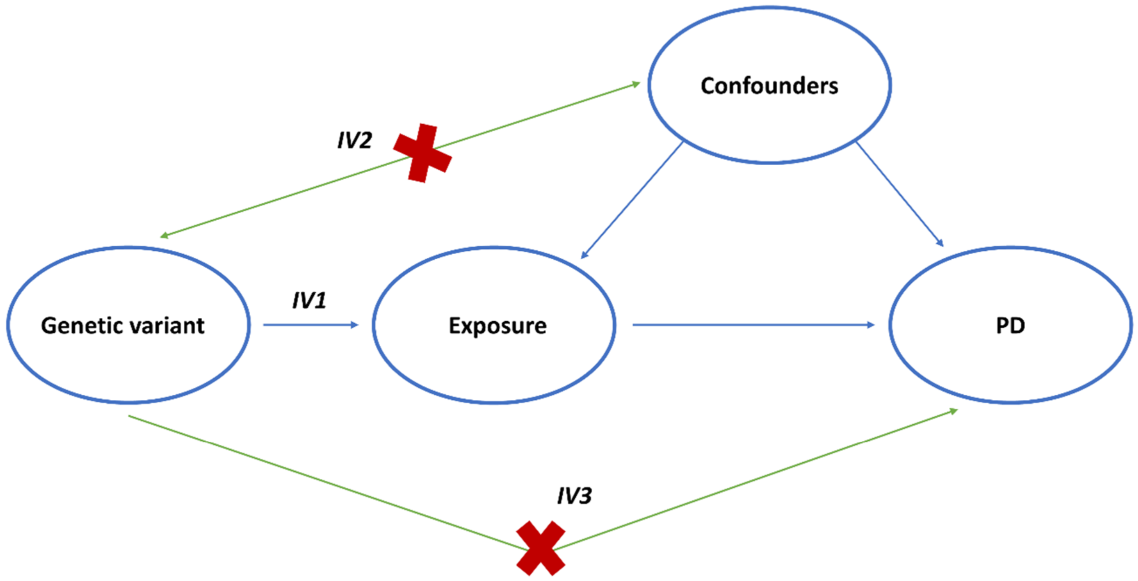

Figure S2. Percentage of articles that addressed specific key methodological questions. Absolute numbers are reported on the top of the bars.

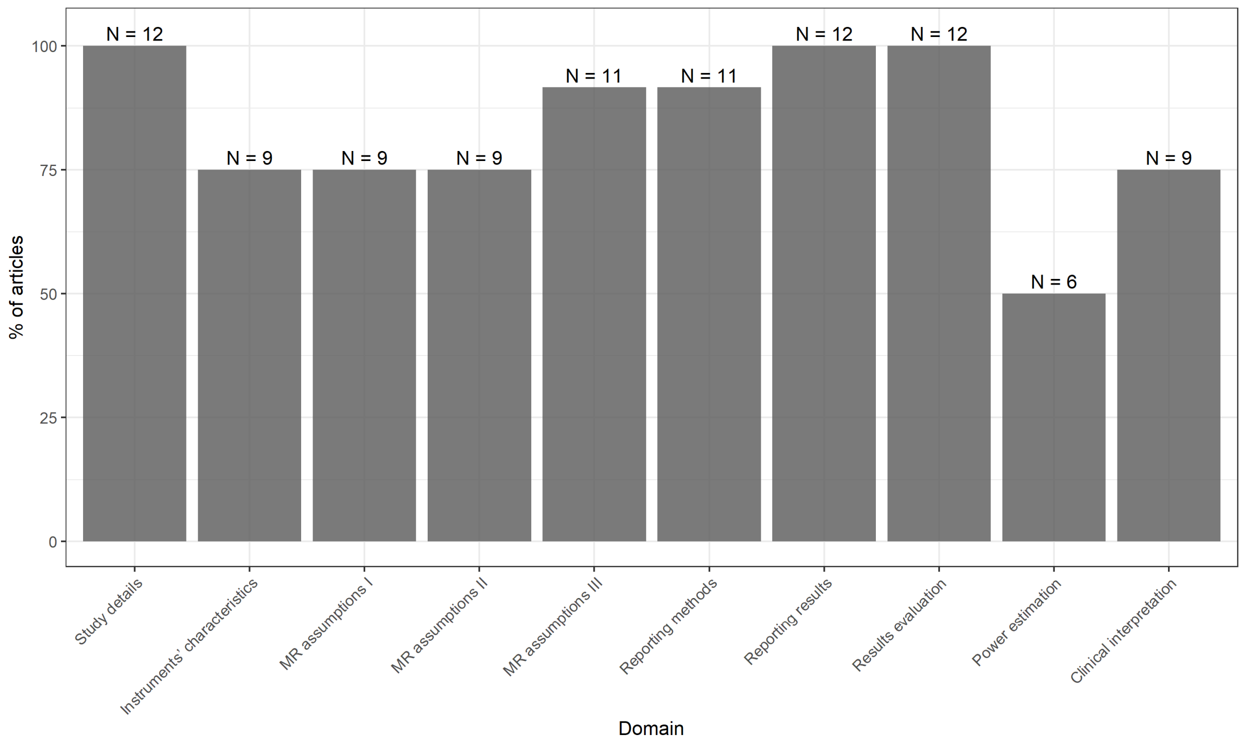

Figure S3: P-values of each exposure.

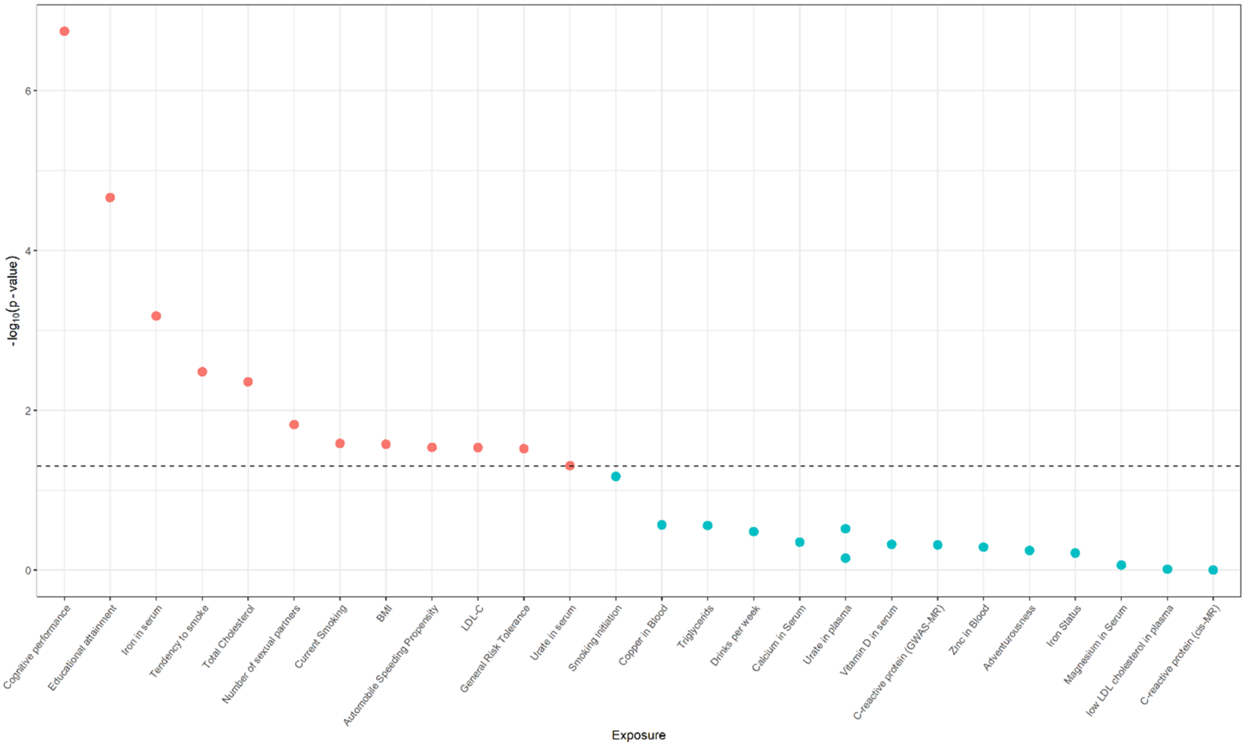

Figure S4: Power estimation for extracted MR studies for different levels of variance explained by the instruments (R^2), varying the sample size, proportion of cases and the odds ratios.

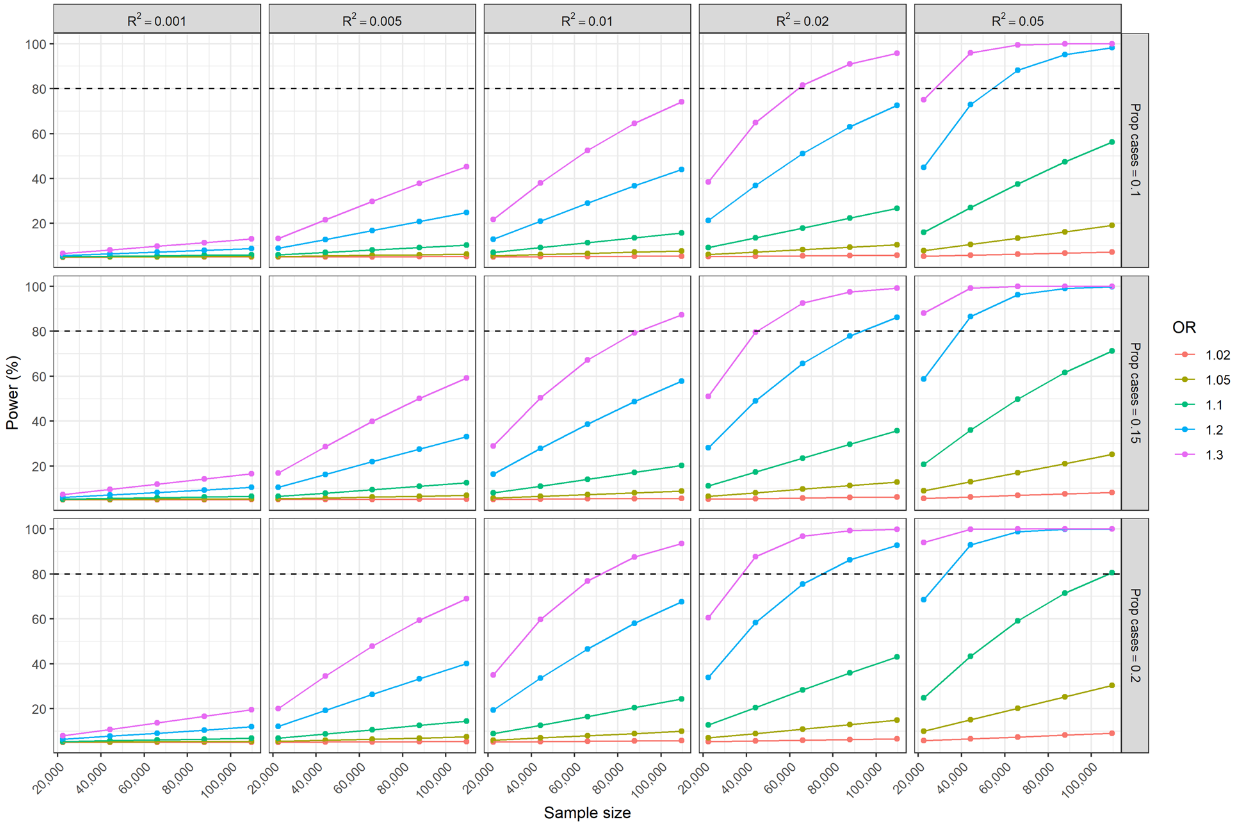

Supplement: Supplementary file 1 — Supplementary figures [file medgen-2022-2139suppa.pdf]
